# Typical Kawasaki disease

**DOI:** 10.1186/1824-7288-41-S2-A47

**Published:** 2015-09-30

**Authors:** Alessandra Marchesi, Isabella Tarissi, Giulia Marucci, Alberto Villani

**Affiliations:** 1 Internal Care Medicine Department, Bambino Gesù Children's Hospital, 00165 Roma, Italy

## 

Kawasaki disease (KD) was first described in Japan in 1967 by Tomisaku Kawasaki [1]. It is an acute, systemic, self-limited vasculitis, whose targets are medium diameter vessels in each organ and apparatus. It is a vasculitis of unknown aetiology, probably multifactorial, that occurs predominantly in infants and young children. The most important complications are coronary arteries aneurysms or ectasia, that develop in 15% to 25% of untreated patients, but only in 5% of patients treated with immunoglobulins within ten days following onset of fever [2,3].

Typical Kawasaki disease is characterized by signs and symptoms which are defined as “diagnostic clinical criteria”, and are:

– ≥5 days of fever

– bilateral non-exudative conjunctivitis

– erythema of the lips and oral mucosa

– changes in the extremities (i.e. erythema of the palms and soles or firm, sometimes painful induration of the hands or feet, erythema of the perineal region)

– cutaneous rash

– cervical lymphadenopathy [3,4].

Diagnosis of KD is based on clinical criteria [3,4]: there are neither typical clinical features nor diagnostic tests. An increasing number of children is reported in literature, having coronary artery aneurysms or ectasia, but who do not fulfill all diagnostic criteria. These cases are defined as *incomplete KD* and *atypical KD.*

Typical KD is diagnosed if:

– fever ≥ 5 days associated with ≥ 4 diagnostic criteria, even before performing echocardiography

– fever ≥ 5 days associated with < 4 diagnostic criteria and possible coronary arteries lesions

– fever at fourth day with ≥ 4 diagnostic criteria and possible coronary arteries lesions.

Finally, other clinical findings may be present in KD:

– Cardiac: coronaritis, pericarditis, myocarditis, endocarditis, mitral regurgitation, aortic and tricuspidal regurgitation (in the acute phase), aortic dilatation (in the later phase), cardiac failure, shock, arrhythmia, coronary artery involvement (in the sub acute phase)

– Vascular: Raynaud phenomenon, peripheral gangrene

– Joints: arthritis or arthralgia

– Nervous System: irritability, aseptic meningitis, sensor-neural hearing loss, transient unilateral peripheral facial nerve palsy

– Gastrointestinal: diarrhoea, vomiting, abdominal pain, acute surgical abdomen, hepatic enlargement and jaundice, acute acalculous distention of the gallbladder (hydrops)

– Urinary: aseptic pyuria, urethritis, testicular swelling

– Skin: erythema and induration at the site of a previous vaccination with Bacille Calmette-Guérin, Beau lines

– Respiratory: cough, rhinorrea, pulmonary nodules and infiltrates.
